# Efficacy of pharmacologic therapies in patients with acute heart failure: A network meta-analysis

**DOI:** 10.3389/fphar.2022.677589

**Published:** 2022-09-23

**Authors:** Hengheng Dai, Haisong Li, Bin Wang, Jingjing Zhang, Ying Chen, Xuecheng Zhang, Yan Liu, Hongcai Shang

**Affiliations:** Dongzhimen Hospital, Beijing University of Chinese Medicine, Beijing, China

**Keywords:** acute heart failure, network meta-analysis, randomized controlled trial, drug therapy, all-cause mortality

## Abstract

**Background:** A network meta-analysis (NMA) of the current recommended drugs for the treatment of acute heart failure (AHF), was performed to compare the relative efficacy.

**Methods:** We used PubMed, EMBASE, Cochrane Clinical Trials Register, and Web of Science systems to search studies of randomized controlled trials (RCT) for the treatment of AHF recommended by the guidelines and expert consensus until 1 December 2020. The primary outcome was all-cause mortality within 30 days. The secondary outcomes included 30-days all-cause rehospitalization, rates of HF-related rehospitalization, rates of adverse events, and rates of serious adverse events. A Bayesian NMA based on random effects model was performed.

**Results:** After screening 14,888 citations, 23 RCTs (17,097 patients) were included, focusing on nesiritide, placebo, serelaxin, rhANP, omecamtiv mecarbil, tezosentan, KW-3902, conivaptan, tolvaptan, TRV027, chlorothiazide, metolazone, ularitide, relaxin, and rolofylline. Omecamtiv mecarbil had significantly lower all-cause mortality rates than the placebo (odds ratio 0.04, 0.01–0.22), rhANP (odds ratio 0.03, 0–0.40), serelaxin (odds ratio 0.05, 0.01–0.38), tezosentan (odds ratio 0.04, 0–0.22), tolvaptan (odds ratio 0.04, 0.01–0.30), and TRV027 (odds ratio 0.03, 0–0.36). No drug was superior to the other drugs for the secondary outcomes and safety outcomes.

**Conclusion:** No drug was superior to the other drugs for the secondary outcomes and safety outcomes. Current drugs for AHF show similar efficacy and safety.

## Introduction

Acute heart failure (AHF) is a clinical syndrome mainly manifested by a marked decrease in myocardial contractility caused by abnormal heart function, an increase in cardiac load, and a sharp decrease in cardiac output (Ponikowski et al., 2016a). The clinical symptoms of AHF include dyspnea, fatigue, and fluid retention, which can adversely affect patient health and quality of life (Arrigo et al., 2020). The mortality caused by AHF during hospitalization in the United States was 4–6% (Adams et al., 2005; Abraham et al., 2008). The readmission rate within 3 months after hospitalization may reach 30% (Ambrosy et al., 2014), and the readmission rate may reach 50% within 4–6 months (Crespo-Leiro et al., 2016). The main drugs recommended for the treatment of AHF include sedatives (i.e., morphine), bronchial antispasmodics (i.e., aminophylline), diuretics (i.e., chlorothiazide), vasodilators (i.e., nesiritide), vasoconstrictors (i.e., epinephrine), and cardiotonics (i.e., dobutamine, Rossignol et al., 2019). However, there are various indicators of the efficacy of the medical treatment of AHF, and there is a lack of comparison of the main outcome indicators in the short term (the evaluation period is either too short or too long). Regarding drug safety, most studies have focused on the number of adverse events and few studies have attempted to determine differences in the incidence of adverse reactions in the treatment of AHF with drugs, especially within 30 days. More importantly, there have been no direct comparisons of AHF-related therapeutic drugs.

AHF is the most common cause of hospitalization in men over 65 years of age (Ponikowski et al., 2016b), and short-term (within 3 months) and long-term (within 1 year) survival rates are not favorable (Chioncel et al., 2017). The pathophysiology of AHF is multifactorial. There are many potential predisposing factors (such as acute coronary syndrome, arrhythmia, kidney damage, and infection), some of which may be related to increased mortality (Arrigo et al., 2017). Despite extensive investigations in prospective randomized trials, no treatment for acute heart failure can prolong survival or reduce mortality (Ferrari et al., 2018). In the absence of treatments to prolong survival or reduce mortality (Ferrari et al., 2018), the main goal of treatment is to stabilize the patient, reduce congestion, relieve symptoms, and reduce organ damage caused by congestion (Harjola et al., 2017). Therefore, to provide a more comprehensive understanding of the impact of treatment on AHF, we reviewed all existing evidence on the medical treatment of AHF, including all drugs recommended by treatment guidelines. We examined all-cause mortality, rates of all-cause rehospitalization, rates of HF-related rehospitalization, rates of adverse events, and the rates of serious adverse events (all outcomes are within 30 days). Among them, all-cause mortality within 30-days was the main efficacy indicator.

## Methods

This study used the Cochrane Collaborative Method (Higgins et al., 2011) and PRISMA Statement (Page et al., 2021), as well as the requirements of the Indirect Treatment Working Group of the International Society for Pharmacoeconomics and Outcomes Research (ISPOR, Massarweh et al., 2021). We registered this study with PROSPERO (registration number: CRD42020169369).

### Data sources and search strategy

We searched the databases of PubMed, EMBASE, Web of Science, and the Cochrane Clinical Trials Register from their starting dates to 1 December 2020, using the following key words: acute decompensated heart failure OR acute heart failure OR heart failure AND All guideline-recommended and expert consensus-recommended drug classes: Diuretics, Vasopressin V2 receptor antagonist, ACEI, ARB, Natriuretic peptide, beta blockers, Ivabradine, Digitalis, levosimendan, and Phosphodiesterase III inhibitor, administered alone. The article search was limited to studies involving human subjects and published in English. The full search strategies for PubMed are provided in Supplementary Appendix file S1.

### Study selection

We excluded the reduplicated studies using Endnote software, and then screened the studies according to their titles or abstracts. Two authors (HHD and HSL) scanned the titles and abstracts of all retrieved articles independently, and irrelevant studies were excluded at this stage (according to inclusion and exclusion criteria). The eligibility of the remaining articles was evaluated for disagreement or uncertainty. Disagreements were resolved by discussion or consensus of a third reviewer.

### Data extraction and quality assessment

Data extraction was performed independently by two authors (HHD and HSL), and the data were checked by a third reviewer. Disagreements were settled by discussion. The following information was extracted from each retrieved article: characteristics of included studies (title, first author, publication year, journal, corresponding address, study design, inclusion, and exclusion criteria, doses of the drugs, treatment duration, and pertinent outcomes). Our outcomes included all-cause mortality, rates of all-cause rehospitalization, rates of HF-related rehospitalization, rates of adverse events, and the rates of serious adverse events (all outcomes are limited to a 30-days period). We appraised study validity according to the risk of bias tool recommended by the Cochrane Collaboration (Higgins et al., 2011). Disagreements were resolved by discussion.

### Data synthesis and analysis

R (version 3.4.2, United States) and GeMTC (version 0.14.3, United States) based on a Bayesian model were used for statistical analysis. For direct comparisons, the standard deviation of random effects and the standard deviation of inconsistency were used to determine whether the study was heterogeneous. The odds ratio (OR) was selected as the statistical quantity for the two-class effect size, and a 95% confidence interval (CI) was used. The network meta-analysis adopts the consistency model, and *p* < 0.05 is considered as statistically significant. The inconsistency test adopts the nodal analysis model (namely, the dot method). A *p* > 0.05 indicates that there is no evidence proving the inconsistency of the study. The convergence of the meshed Meta was tested using the potential scale reduction parameter (PSRF). IA PSRF close to one means that the convergence of the study is good and the conclusions drawn by the Meta analysis are reliable. Each analysis was based on vague priors for effect sizes (to yield results that are similar to conventional statistical analysis). We used deviance and the deviance information criterion to appraise model fit. We report results of network meta-analysis as odds ratios with 95% credible intervals for categorical outcomes and weighted mean differences with 95% credible intervals for continuous outcomes.

### Inclusion criteria and exclusion criteria

The inclusion criteria for the studies were as follows: 1) the study was a randomized, controlled trial (RCT); 2) the study included participants who were adult patients with AHF; 3) the two groups of intervention measures were single drug therapy (medicine for AHF); and 4) the trial had relevant outcomes (occurring within 30 days) including all-cause mortality, rates of all-cause rehospitalization, rates of HF-related rehospitalization, rates of adverse events, and rates of serious adverse events. The exclusion criteria were as follows: 1) observational studies; 2) studies on CHF or failure to report expected results; 3) comparative studies between different doses of the same drug; and 4) observations on the efficacy of drug combinations (> one drug).

## Results

### Study characteristics and quality

**FIGURE 1:**
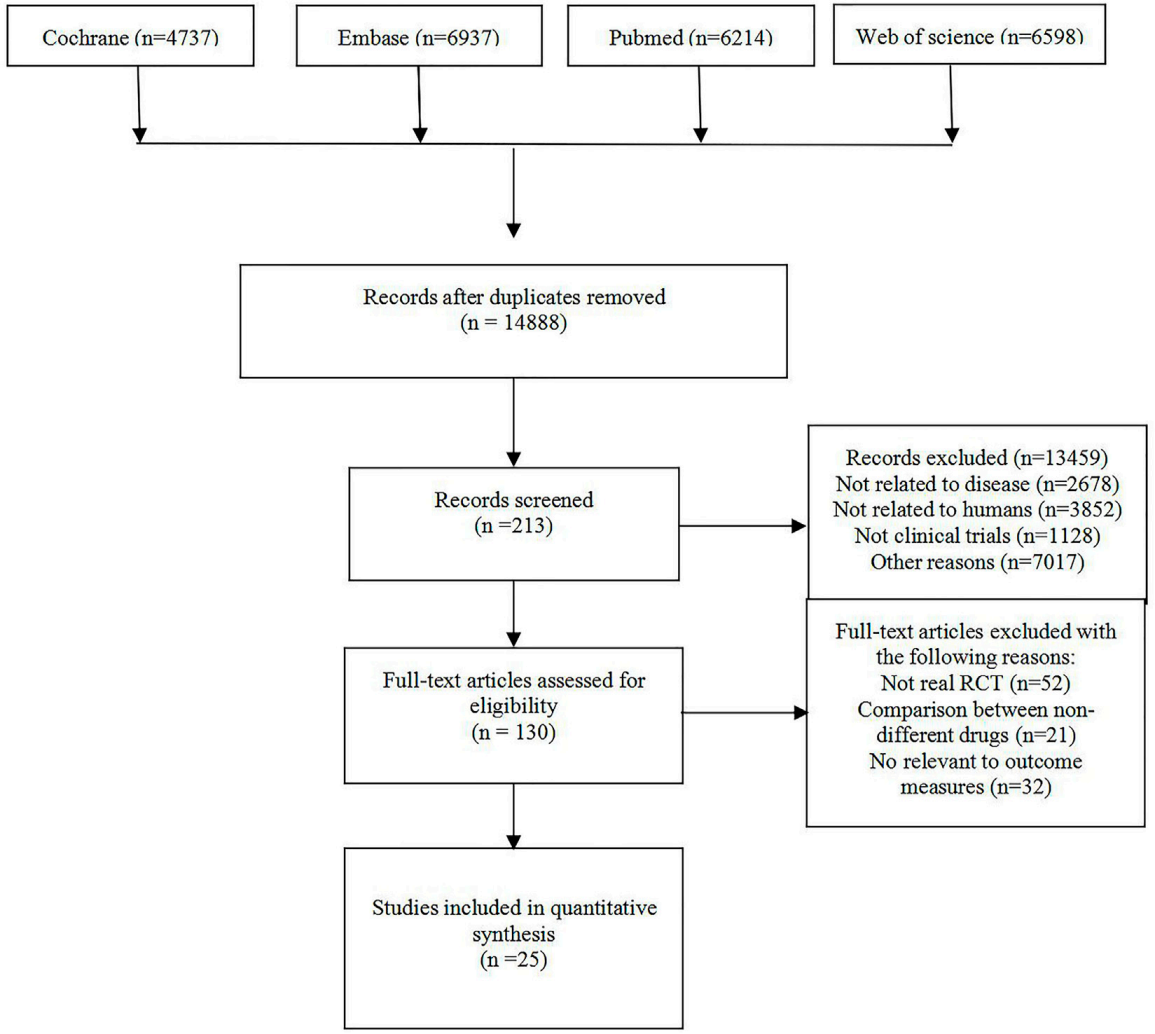
Review profile. RCT = randomized controlled trial.

**FIGURE 2:**
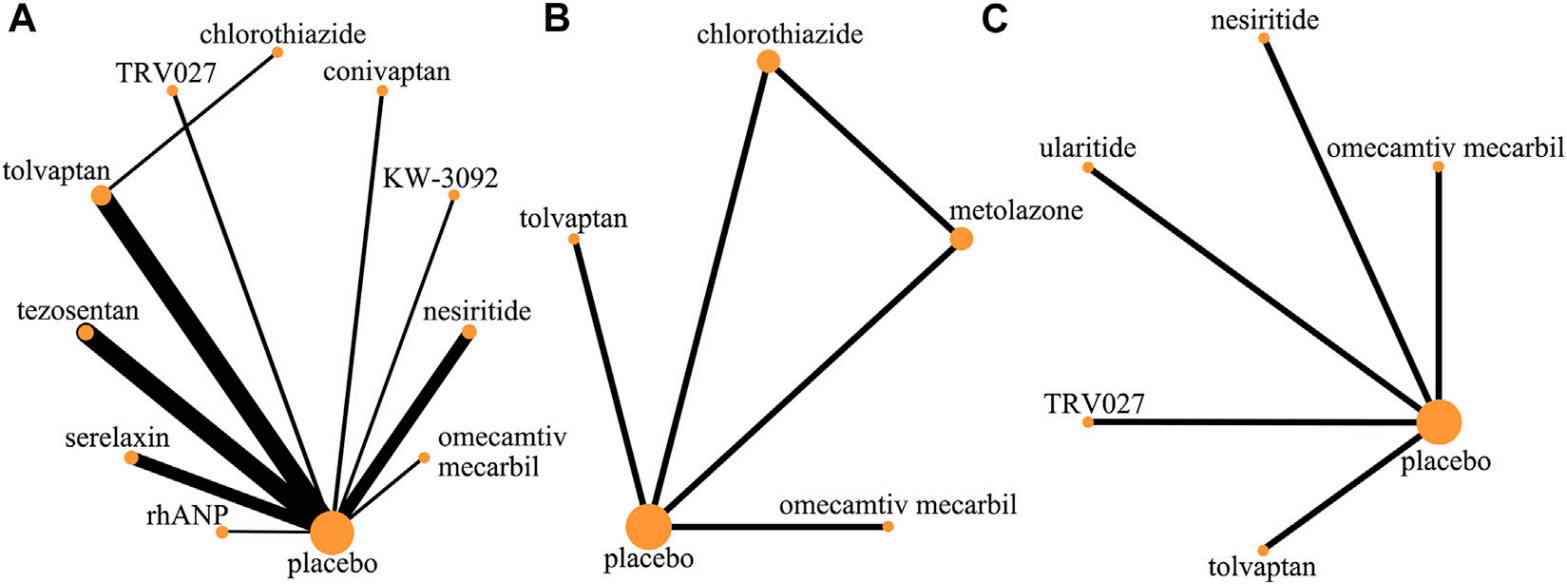
Evidence network (death and rehospitalization). **(A)** All-cause mortality within 30 days; **(B)** all-cause readmission rate within 30 days; **(C)** HF-related readmission rate within 30 days.

**FIGURE 3:**
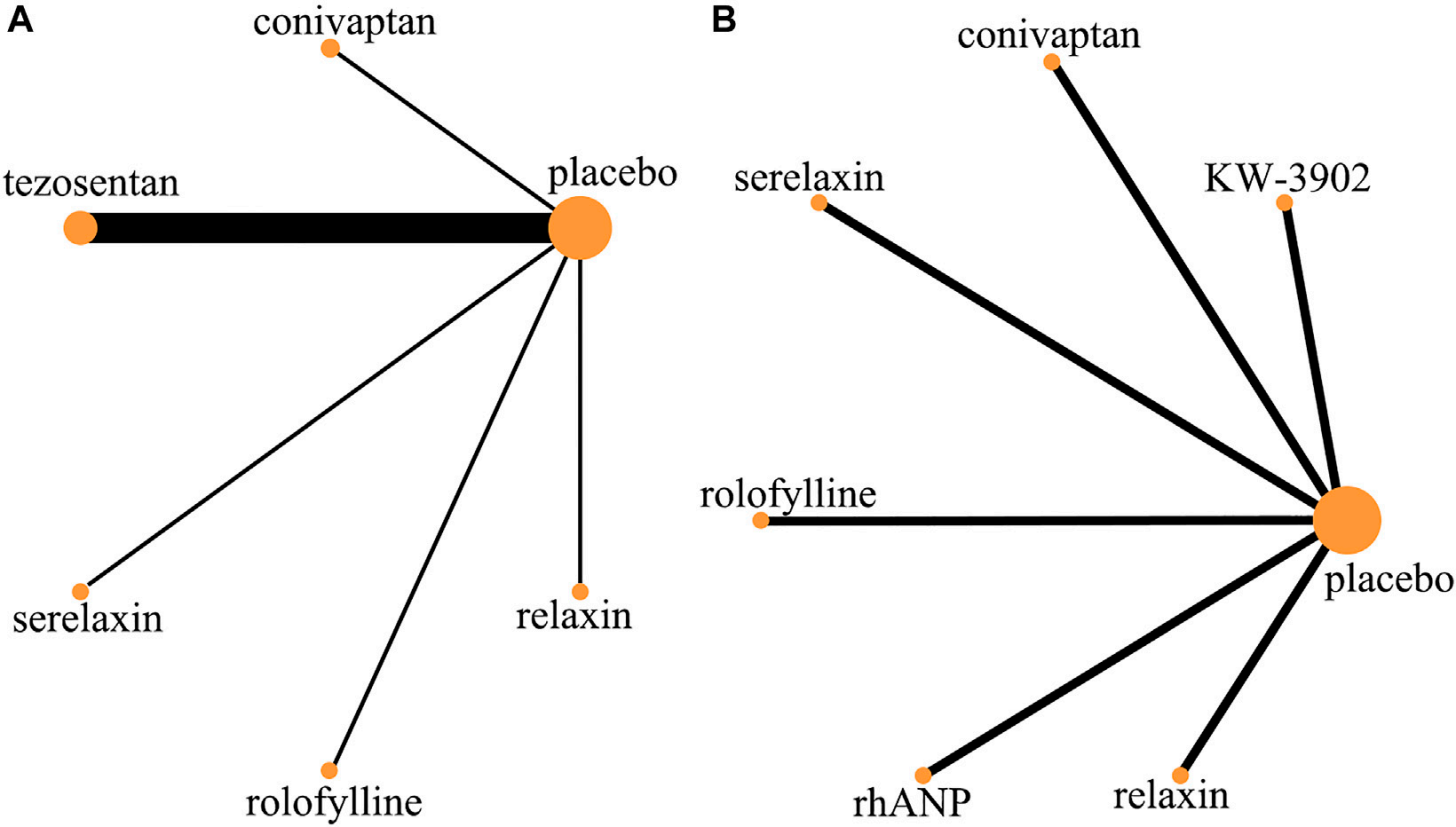
Evidence network (adverse event). **(A)** Rate of adverse event; **(B)** rate of serious adverse event.

After screening 14,888 citations (Figure 1), we included 23 studies, with a total of 17,097 patients, treated with diuretics (conivaptan, tolvaptan, metolazone, chlorothiazide, KW-3902, rolofylline), vasodilators (nesiritide, rhANP, urapidil, ularitide, relaxin, serelaxin, tezosentan, TRV027, nitroglycerin), cardiotonics (levosimendan, dobutamine, omecamtiv mecarbil) or placebo (Table 1). Patients had a median age of 67.5 years and 66% were males. Patients were followed for a median of 2.7 months. Some drugs in the study could not be linked to other study drugs, so they were not included in the analysis (Figure 2 and Figure 3). All trials had a low risk of bias according to Cochrane metrics (Supplementary Appendix Figure AS1). Only two trials directly compared one drug to another, which means that only a comparison with a placebo could provide direct evidence. Therefore, the effects of drugs could only be compared with each other using indirect evidence.

**TABLE 1:** Key features of the included studies.

Trial	Phase	Patients	Experimental group	Control group	Age (years)	Male (%)	Follow-up (months)	Outcomes reported^a^
Miller et al. (2008)	II	101	Nesiritide	Placebo	55.7	65	1	1
Teerlink et al. (2009)	II	229	Relaxin	Placebo	70.3	56	4	4, 5
O’Connor et al. (2011)	III	7,007	Nesiritide	Placebo	67	66	1	1, 3
Teerlink et al. (2013)	II	1,161	Serelaxin	Placebo	72	62	6	1
Ponikowski et al. (2014)	II	71	Serelaxin	Placebo	69	75	1	1,4,5
Wang et al. (2016)	III	476	RhANP	Placebo	55	75	1	1,5
Wang et al. (2017)	II	58	Urapidil	Nitroglycerin	76	57	1	1,3
Packer et al. (2017)	III	2,157	Ularitide	Placebo	69	66	6	3
Yang et al. (2017)	II	180	Urapidil	Nitroglycerin	77	58	1	1,2
Bergh et al. (2010)	Ⅳ	30	Levosimendan	Dobutamine	71	85	1	1,2,4
Mebazaa et al. (2009)	III	1,327	Levosimendan	Dobutamine	66	72	6	1
Teerlink et al. (2016)	II	606	Omecamtiv mecarbil	Placebo	66	77	6	1,2,3
Cotter et al. (2004)	II	129	Tezosentan	Placebo	70	67	1	1,4
Torre-Amione et al. (2003)	III	285	Tezosentan	Placebo	61	79	6	1,4
McMurray et al. (2007)	III	1,435	Tezosentan	Placebo	70	60	6	1,4
Givertz et al. (2007)	III	146	KW-3902	Placebo	67	68	1	1,5
Goldsmithet al. (2008)	II	162	Conivaptan	Placebo	63	63	1	1,4,5
Cotter et al. (2008)	III	301	Rolofylline	Placebo	71	59	2	4,5
Shanmugam et al. (2016)	II	51	Tolvaptan	Placebo	58	71	1	1
Felker et al. (2017)	III	257	Tolvaptan	Placebo	65	66	1	1,2
Pang et al. (2017)	II	618	TRV027	Placebo	70	62	6	1,3
Konstam et al. (2017)	III	250	Tolvaptan	Placebo	68	74	1	1,2
Cox et al. (2020)	Ⅳ	60	Metolazone or Chlorothiazide	Tolvaptan	62	71	1	1,2,4

a 1 = death; 2 = rehospitalization; 3 = rehospitalization for HF; 4 = rate of adverse events; 5 = rate of serious adverse events.

### Effect of drugs on mortality within 30 days

In all-cause mortality within 30 days, a total of 19 trials (3 trials could not be compared with other drugs), 10 drugs (including one diuretic, three vasodilators, and five vasoconstrictors, and one cardiotonic agent), and 12,777 patients were involved (Table 2). The comparison of the results of the consistency model and the inconsistency model showed that the results were heterogeneous but the sensitivity analysis that excludes one by one showed that the results were stable. The results showed that omecamtiv mecarbil had significantly lower all-cause mortality rates than the placebo (odds ratio 0.04, 0.01–0.22), rhANP (odds ratio 0.03, 0–0.40), serelaxin (odds ratio 0.05, 0.01–0.38), tezosentan (odds ratio 0.04, 0–0.22), tolvaptan (odds ratio 0.04, 0.01–0.30), and TRV027 (odds ratio 0.03, 0–0.36). Conivaptan, KW-3902, and nesiritide had significantly higher all-cause mortality rates than omecamtiv mecarbil. The pairwise comparison results of the other drugs were not significant. Generally, the effects on all-cause mortality within 30 days, from low to high, were omecamtiv mecarbil, conivaptan, KW-3902, chlorothiazide, nesiritide, tezosentan, serelaxin, rhANP, tolvaptan, and TRV027. Among them, omecamtiv mecarbil, conivaptan, and KW-3902 may have a lower impact on all-cause mortality within 30 days than the placebo, while chlorothiazide, nesiritide, tezosentan, serelaxin, rhANP, tolvaptan, and TRV027 may have a higher impact than the placebo (Supplementary Appendix Figure AS2).

**TABLE 2:** Direct comparisons among different drugs of all-cause mortality within 30 days (reported as point estimates of odds ratios or weighted mean differences with 95% credible intervals, with number of studies contributing to network). Among them, those marked as red are diuretics, those marked as yellow are vasodilators, those marked as blue are cardiotonics, those marked as green are vasoconstrictors, and those marked as white are placebos.

Chlorothiazide										
0.21 (0,20.18)	Conivaptan									
0.33 (0,35.05)	1.43 (0.04,82.22)	KW-3902								
1.82 (0.04,99.79)	7.16 (0.57,259.48)	4.58 (0.38,145.00)	Nesiritide							
30.45 (0.46,1723.89)	126.74 (7.16,5312.09)	83.57 (4.33,2696.85)	18.72 (1.70,116.27)	Omecamtiv mecarbil						
1.29 (0.03,50.79)	5.07 (0.46,135.28)	3.20 (0.28,85.35)	0.77 (0.16,1.83)	0.04 (0.01,0.22)	Placebo					
0.95 (0.01,66.97)	4.23 (0.19,168.61)	2.52 (0.12,114.96)	0.61 (0.04,3.77)	0.03 (0,0.40)	0.81 (0.10,4.77)	RhANP				
1.46 (0.02,69.37)	5.89 (0.39,217.88)	3.82 (0.24,112.89)	0.87 (0.10,3.70)	0.05 (0.01,0.38)	1.15 (03.30,3.94)	1.48 (0.15,15.86)	Serelaxin			
1.07 (0.02,44.37)	4.32 (0.30,140.97)	2.79 (0.18,88.33)	0.70 (0.07,1.97)	0.04 (0,0.22)	0.91 (0.24,1.92)	1.08 (0.11,8.88)	0.77 (0.12,3.41)	Tezosentan		
1.21 (0.03,43.40)	5.16 (0.38,141.92)	3.30 (0.24,99.64)	0.76 (0.11,2.75)	0.04 (0.01,0.30)	1.01 (0.32,2.87)	1.26 (0.14,12.99)	0.87 (0.17,4.72)	1.16 (0.29,6.10)	Tolvaptan	
1.09 (0.02,51.04)	4.25 (0.25,160.53)	2.88 (0.16,111.14)	0.61 (0.06,3.80)	0.03 (0,0.36)	0.81 (0.17,4.09)	1.02 (0.10,15.56)	0.70 (0.10,6.01)	0.91 (0.19,8.21)	0.81 (0.13,5.90)	TRV027

### Effect of drugs on the rates of rehospitalization within 30 days

In the rate of all-cause rehospitalization within 30 days, there are five trials (2 trials could not be compared with other drugs), five drugs (including two diuretics, one vasoconstrictor, and one cardiotonic), and 1,163 patients were involved (Table 3 and Table 4). Comparison of the consistency model and the inconsistency model results showed good agreement (0.23 vs. 0.22). Although the results of the pairwise comparison of the drugs were not statistically significant, the effects on the rates of all-cause rehospitalization within 30 days, from low to high, were chlorothiazide, metolazone, omecamtiv mecarbil and tolvaptan. Among them, the effect of chlorothiazide and metolazone on the rate of all-cause rehospitalization within 30 days may be lower than that of placebo, while the effect of omecamtiv mecarbil and tolvaptan may be higher than that of placebo. In the rates of HF-related rehospitalization within 30 days, a total of six trials (one trial could not be compared with other drugs), five drugs (including one vasodilator, two vasoconstrictors, and two cardiotonics), and 10,473 patients were involved. Comparison of the consistency model and the inconsistency model results showed good agreement (0.24 vs. 0.23). There was no statistical difference in the impact of these five drugs on the rates of HF-related rehospitalization within 30 days. The risk probabilities, from low to high, were TRV027, tolvaptan, omecamtiv mecarbil, nesiritide, and ularitide. Among them, the effects of TRV027 and tolvaptan on the rate of HF-related rehospitalization within 30 days may be lower than the placebo, whereas the effects of omecamtiv mecarbil, nesiritide, and ularitide may be higher than placebo.

**TABLE 3:** Direct comparisons between different drugs of all-cause readmission rate within 30 days (reported as point estimates of odds ratios or weighted mean differences with 95% credible intervals, with number of studies contributing to network). Among them, those marked as red are diuretics, those marked as blue are cardiotonics, those marked as green are vasoconstrictors, and those marked as white are placebos.

Chlorothiazide				
1.61 (0.36,8.07)	Metolazone			
1.56 (0.31,7.77)	0.96 (0.19,4.88)	Omecamtiv mecarbil		
1.27 (0.28,5.35)	0.77 (0.17,3.42)	0.81 (0.40,1.60)	Placebo	
1.02 (0.18,5.66)	0.62 (0.11,3.66)	0.66 (0.22,1.87)	0.82 (0.34,1.82)	Tolvaptan

**TABLE 4:** Direct comparisons between different drugs of HF-related readmission rate within 30 days (reported as point estimates of odds ratios or weighted mean differences with 95% credible intervals, with number of studies contributing to network). Among them, those marked as yellow are vasodilators, those marked as blue are cardiotonics, those marked as green are vasoconstrictors, and those marked as white are placebos.

Nesiritide					
0.83 (0.30,2.32)	Omecamtiv mecarbil				
0.97 (0.52,1.82)	1.17 (0.50,2.70)	Placebo			
1.17 (0.32,4.35)	1.40 (0.35,5.92)	1.20 (0.40,3.85)	Tolvaptan		
0.59 (0.19,1.70)	0.71 (0.20,2.41)	0.60 (0.23,1.43)	0.50 (0.11,2.08)	TRV027	
0.97 (0.38,2.39)	1.16 (0.41,3.34)	0.99 (0.51,1.90)	0.82 (0.22,3.01)	1.64 (0.55,5.19)	Ularitide

### Effect of drugs on the rates of adverse events within 30 days

In the rates of adverse events within 30 days, a total of nine trials (2 trials were not comparable with other drugs), five drugs (including two vasodilators and three vasoconstrictors), and 2,725 patients were involved (Table 5 and Table 6). Comparison of the consistency model and the inconsistency model results showed good agreement (0.60 vs. 0.54). There was no statistical difference in the effects of these five drugs on the rates of adverse events within 30 days. The risk probabilities, from low to high, were conivaptan, tezosentan, serelaxin, relaxin, and rolofylline. Among them, conivaptan, tezosentan, and serelaxin may have a lower impact on the rates of adverse events within 30 days than the placebo, while the impact of relaxin and rolofylline may be higher than the placebo. In the rates of serious adverse events within 30 days, a total of six trials, six drugs (including three vasodilators and three vasoconstrictors), and 1,386 patients were involved. Comparison of the consistency model and the inconsistency model results showed that they were in good agreement (0.49 vs. 0.48). Similar to the rates of adverse events, there was no statistical difference between the effects of drugs on the rates of adverse events. The risk probability, from low to high, was serelaxin, rhANP, KW-3902, rolofylline, relaxin, and conivaptan. Among them, serelaxin and rhANP may have a higher impact on the incidence of serious adverse events within 30 days than the placebo, while KW-3902, rolofylline, relaxin, and conivaptan may have a lower impact than the placebo (Supplementary Appendix Figure AS3).

**TABLE 5:** Direct comparisons between different drugs of the rate of adverse event within 30 days (reported as point estimates of odds ratios or weighted mean differences with 95% credible intervals, with number of studies contributing to network). Among them, those marked as yellow are vasodilators, those marked as green are vasoconstrictors, and those marked as white are placebos.

Conivaptan					
3.03 (0.65,15.39)	Placebo				
5.18 (0.62,46.47)	1.68 (0.38,7.83)	Relaxin			
3.99 (0.47,37.03)	1.31 (0.31,5.71)	0.78 (0.09,6.35)	Rolofylline		
6.03 (0.60,62.35)	1.95 (0.38,10.54)	1.18 (0.12,10.99)	1.51 (0.15,14.58)	Serelaxin	
2.48 (0.43,15.82)	0.82 (0.35,1.91)	0.48 (0.08,2.70)	0.63 (0.11,3.37)	0.41 (0.06,2.74)	Tezosentan

**TABLE 6:** Direct comparisons between different drugs in the rate of serious adverse events within 30 days (reported as point estimates of odds ratios or weighted mean differences with 95% credible intervals, with number of studies contributing to network). Among them, those marked as yellow are vasodilators, those marked as green are vasoconstrictors, and those marked as white are placebos.

Conivaptan						
0.62 (0.07,4.57)	KW-3902					
0.93 (0.21,4.12)	1.56 (0.38,6.74)	Placebo				
0.93 (0.14,7.69)	1.63 (0.24,12.60)	1.08 (0.27,4.38)	Relaxin			
0.47 (0.01,6.22)	0.78 (0.02,9.82)	0.52 (0.02,4.24)	0.46 (0.01,5.94)	RhANP		
1.27 (0.16,8.34)	2.21 (0.30,14.34)	1.39 (0.34,4.90)	1.26 (0.16,8.47)	2.53 (0.20,85.77)	Rolofylline	
3.01 (0.28,33.06)	5.32 (0.52,53.45)	3.31 (0.52,20.60)	3.03 (0.29,27.69)	6.17 (0.37,266.00)	2.55 (0.25,25.30)	Serelaxin

## Discussion

The results of the study showed that, for all-cause mortality within 30 days, omecamtiv mecarbil was somewhat better than most of the other drugs that we included. Omecamtiv mecarbil is a myosin exercise activator. It can directly act on cardiac myosin, resulting in increased strength of the heart muscle and prolonged contraction time (Nanasi et al., 2018). However, there was only one RCT study involving omecamtiv mecarbil that met our inclusion criteria and the results of the study may be biased. Within 30 days, the rates of all-cause rehospitalization, the rates of HF-related rehospitalization, the rates of adverse events, and the rates of serious adverse events, showed no significant differences associated with the effects of each drug, which was the same as the previous results (Curfman 2019). The difference is that we have provided the risk probability of each drug to the research outcome and this has reference value for clinicians. During the process of screening related studies, we found that the drugs reported for treating AHF in the RCT studies mainly include vasoconstrictors, vasodilators, cardiotonics and diuretics. However, only a few studies reported the rate of rehospitalization or mortality within 30 days. Most studies focused on specific indicators to determine the efficacy of the drug and did not report key outcome indicators. For adverse events, most studies reported the number of adverse events but did not report the incidence and this made our study more difficult.

Our analysis was limited by the data in the research and the data structure of the reports. Our criteria for inclusion and exclusion were relatively strict, resulting in a relatively small number final number of documents. Because of this, caution is needed when interpreting the results. Most of the studies we included were efficacy comparisons between drugs and placebo. There are few direct comparisons between drugs and this certainly impacted the final results. Although we have confidence in our search strategy, some experiments, such as some non-English language experiments, were excluded. The results are also limited by modeling assumptions. Notably, the present meta-analyses did not include studies of sodium glucose co-transporter-2 inhibitors (SGLT2i). SGLT2i have recently been recommended as a first-line foundational treatment for chronic heart failure. SGLT2i (Sotagliflozin) was also found to improve acute heart failure prognosis in the SOLOIST trial (Bhatt et al., 2021) and is being further investigated in the EMPULSE trial (empagliflozin for acute heart failure, Voors et al., 2022). It is reasonable to believe that SGLT2i may decrease cardiovascular mortality and heart failure readmissions regardless of heart failure acuity. These benefits could potentially stem from the following mechanisms: 1) diuretic and natriuretic effects (including inhibition of the Na +/H + exchanger, Griffin et al., 2020); 2) restoration of myocardial energetics (Garcia-Ropero et al., 2019); 3) improvement in diastolic heart function (Santos-Gallego et al., 2020); 4) improvement in aortic stiffness and systemic vascular resistance (Requena-Ibáñez et al., 2021), and 5) inhibition of the NHE (Uthman et al., 2018). As the evidence for SGLT2i for heart failure outcomes is evolving and their therapeutic mechanisms are being better elucidated, future meta-analyses should include studies of SGLT2i.

## Conclusion

We analyzed clinical trials that included all drugs recommended by the guidelines for the treatment of AHF and used Bayesian network meta-analysis to compare drug efficacy. Omecamtiv mecarbil provided a greater reduction in all-cause mortality within 30 days, compared with the other drugs. Within 30 days, the rates of all-cause rehospitalization, rates of HF-related rehospitalization, rates of adverse events, and rates of serious adverse events were all similar among the various drugs. The individual advantages of these drugs cannot be determined with currently available data. A more comprehensive comparison of individual drugs is needed to determine if any of these drugs provide significant advantages. AHF is a life-threatening condition. It is important to merge data or compare outcomes between RCTs that use different interventions. However, a comparison and meta-analyses were hampered because of the heterogeneity of outcome reporting in systematic reviews (Kewcharoen et al., 2019). To improve the consistency of outcomes, it is necessary to develop a core outcome set (COS) for AHF in future studies.
