# Supplementary material for: Efficacy of pharmacologic therapies in patients with acute heart failure: A network meta-analysis
Source: Front Pharmacol. 2022 Sep 23;13:677589. doi: 10.3389/fphar.2022.677589 (PMC9537610; doi:10.3389/fphar.2022.677589)
Supplement: Supplementary file 1 [file Table1.DOCX]

**Appendix**

**File 1**

Pubmed: Search (((“acute heart failure syndrome”[Title/Abstract] OR “acute left heart failure”[Title/Abstract] OR “acute right heart failure” [Title/Abstract] OR “acute total failure”[Title/Abstract] OR “heart failure”[Title/Abstract])))

AND

((Diuretics[Title/Abstract] OR “Myeloid diuretics”[Title/Abstract] OR Furosemide[Title/Abstract] OR “Etacrynic acid”[Title/Abstract] OR Bumetanide [Title/Abstract] OR Piretanide[Title/Abstract] OR Torasemide[Title/Abstract] OR “Thiazide diuretics/Thiazide diuretics agent”[Title/Abstract] OR Hydrochlorothiazide [Title/Abstract] OR indapamide[Title/Abstract] OR metolazone[Title/Abstract] OR “Potassium-preserving diuretics”[Title/Abstract] OR Amiloride[Title/Abstract] OR Ampicillin[Title/Abstract] OR Spironolactone[Title/Abstract] OR “Aldosterone antagonist/Mineralocorticoid Receptor Antagonists”[Title/Abstract] OR Aldosterone[Title/Abstract] OR eplerenone[Title/Abstract] OR “Vasopressin V2 receptor antagonist”[Title/Abstract] OR Tolvaptan[Title/Abstract] OR “Angiotensin converting enzyme inhibitor/dipeptidyl carboxypeptidase inhibitor”[Title/Abstract] OR Captopril[Title/Abstract] OR Enalapril[Title/Abstract] OR Lisinopril[Title/Abstract] OR Perindopril[Title/Abstract] OR Ramipril[Title/Abstract] OR Fosinopril[Title/Abstract] OR Quinapril[Title/Abstract] OR Benazepril[Title/Abstract] OR “angiotensin II receptor antagonist/angiotensin receptor antagonist”[Title/Abstract] OR Candesartan[Title/Abstract] OR valsartan[Title/Abstract] OR losartan[Title/Abstract] OR “sacurbactra valsartan”[Title/Abstract] OR Irbesartan[Title/Abstract] OR Telmisartan[Title/Abstract] OR Olmesartan[Title/Abstract] OR “Natriuretic peptide/atrial natriuretic factor”[Title/Abstract] OR “beta blockers/beta adrenergic receptor blocking agent”[Title/Abstract] OR “Metoprolol succinate”[Title/Abstract] OR Metoprolol[Title/Abstract] OR Betaloc[Title/Abstract] OR bisoprolol[Title/Abstract] OR carvedilol[Title/Abstract] OR Ivabradine[Title/Abstract] OR Digitalis[Title/Abstract] OR Digoxin[Title/Abstract] OR Celandine/Chelidonium[Title/Abstract] OR strophanthin-K/strophanthin[Title/Abstract] OR levosimendan[Title/Abstract] OR “Phosphodiesterase III inhibitor”[Title/Abstract] OR Amrinone[Title/Abstract] OR Milinone[Title/Abstract]))

“randomized controlled trial”[Publication Type] OR randomized[Title/Abstract] OR placebo[Title/Abstract]


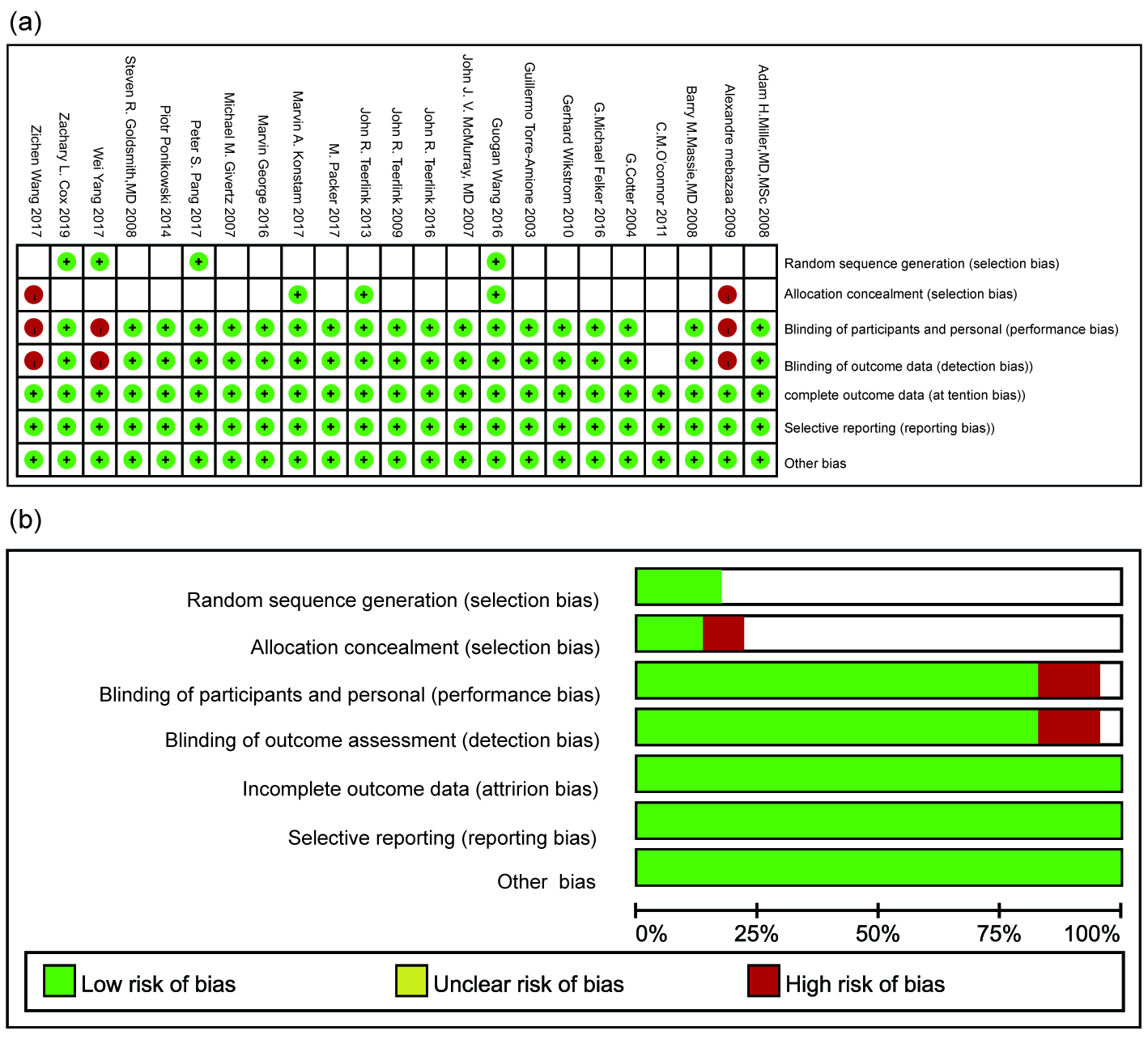


**Fig S1.** Bias risk assessment of included studies. (a) Risk of bias summary. (b) Risk of bias graph.


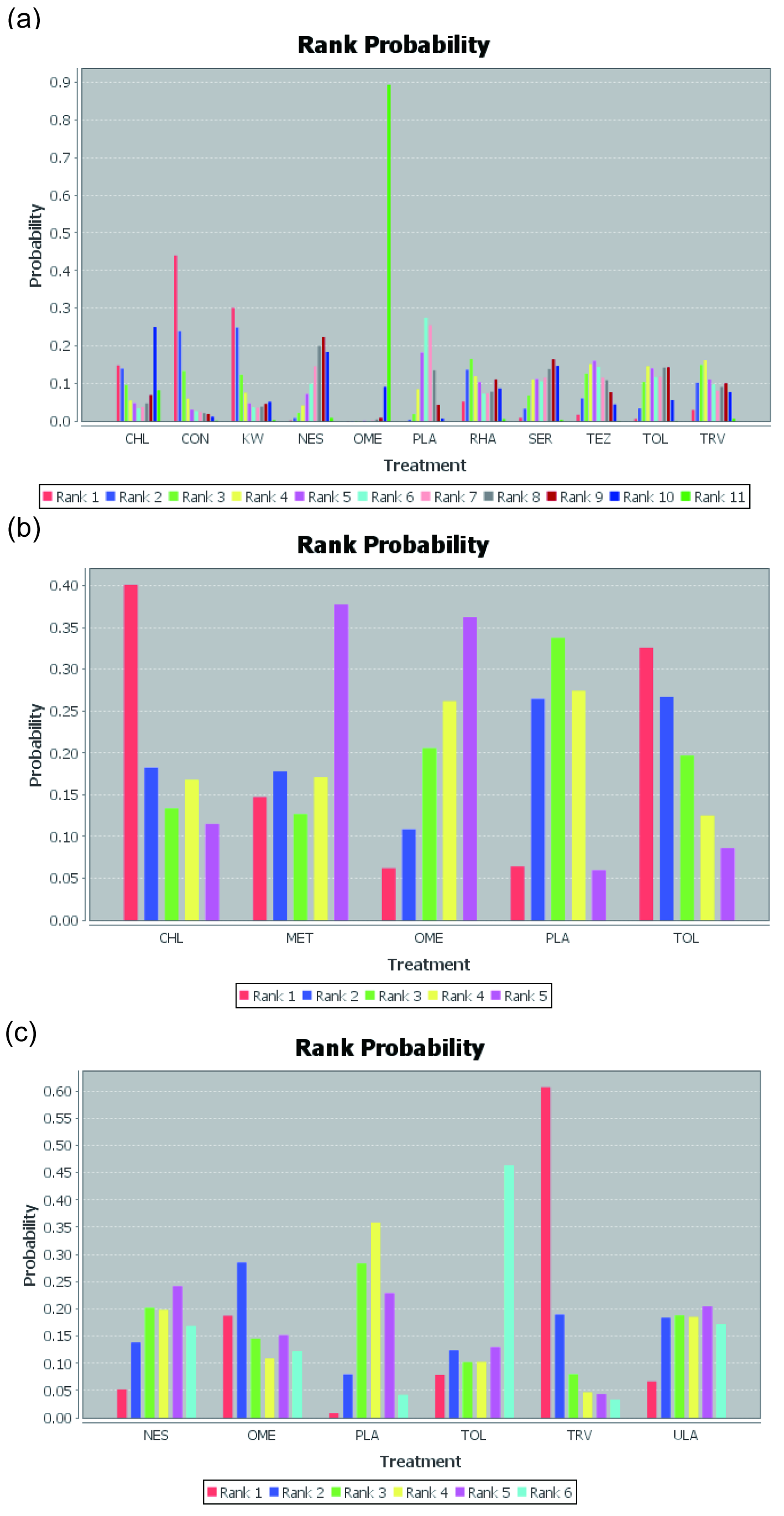


**Fig S2.** Rank probability (death and rehospitalization). OME=omecamtiv mecarbil, CON=conivaptan, KW=KW-3902, PLA=placebo, CHL=chlorothiazide, NES=nesiritide, TEZ=tezosentan, SER=serelaxin, RHA=rhANP, TOL=tolvaptan, TRV=TRV027, MET=metolazone, ULA=ularitide. (a) all-cause mortality within 30 d. (b) All-cause readmission rate within 30 d. (c) HF-related readmission rate within 30 d.


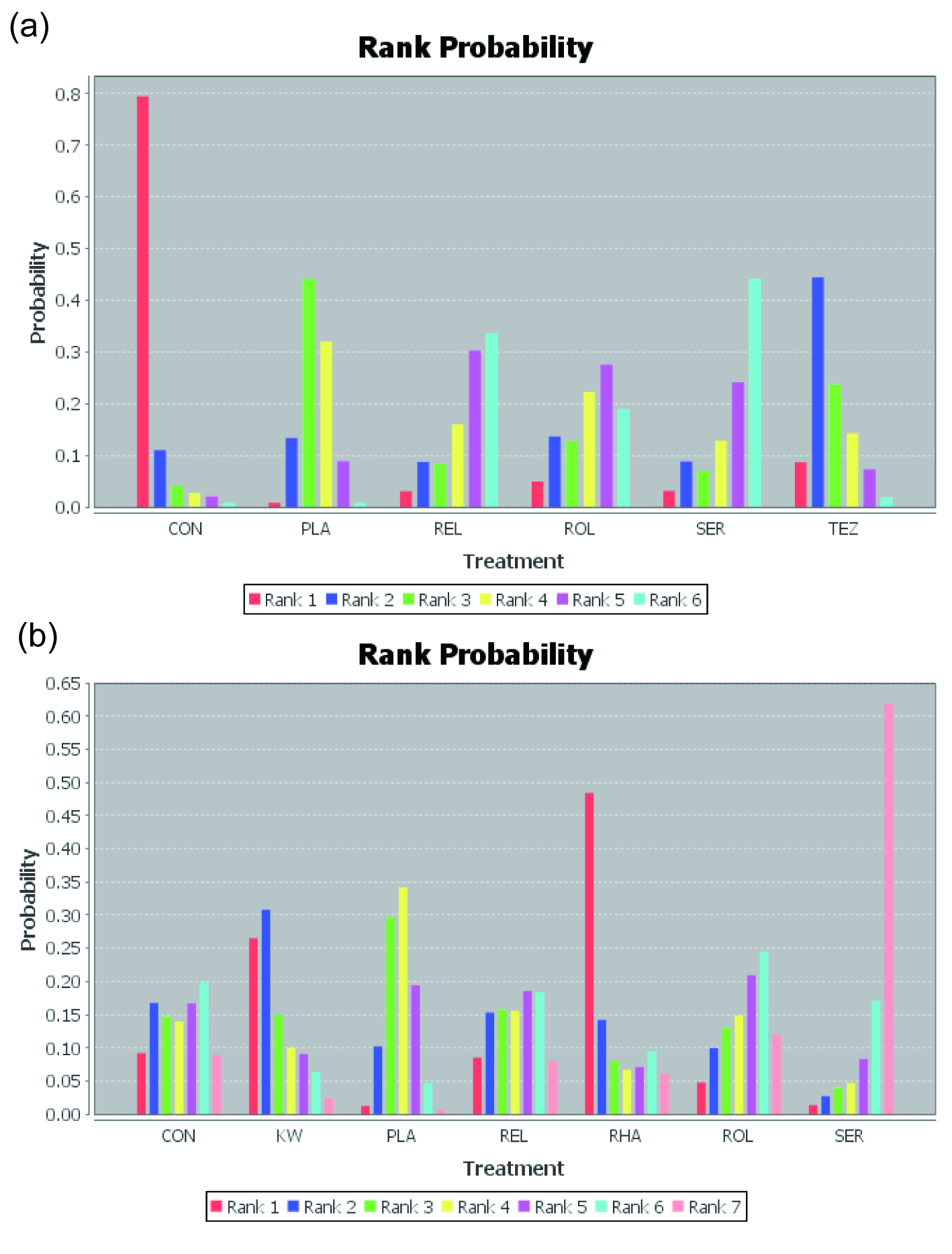


**Fig S3.** Rank probability (adverse event). CON=conivaptan, PLA=placebo, REL=relaxin, ROL=rolofylline, SER=serelaxin, TEZ=tezosentan, KW=KW-3902, RHA=rhANP. (a) Rate of adverse event. (b) Rate of serious adverse event.
